# SpoIVA is an essential morphogenetic protein for the formation of heat- and lysozyme-resistant spores in *Clostridium sporogenes* NBRC 14293

**DOI:** 10.3389/fmicb.2024.1338751

**Published:** 2024-04-24

**Authors:** Ritsuko Kuwana, Bruno Dupuy, Isabelle Martin-Verstraete, Hiromu Takamatsu

**Affiliations:** ^1^Faculty of Pharmaceutical Sciences, Setsunan University, Hirakata, Osaka, Japan; ^2^Laboratoire Pathogenese des Bacteries Anaerobies, Institut Pasteur, Paris, France; ^3^Institut Universitaire de France, Paris, France

**Keywords:** *Clostridium sporogenes*, sporulation, spore, spore coat, cortex, morphogenetic protein, *spoIVA*

## Abstract

*Clostridium sporogenes* is an anaerobic spore-forming bacterium genetically related to *Clostridium botulinum* but lacks toxin genes. The sporulation mechanism and spore structures of anaerobic bacteria, including *C. sporogenes*, have not been comprehensively analyzed. Based on 16S rRNA gene analysis, it has been determined that *C. sporogenes* NBRC 14293 belongs to *C. botulinum* Group I. Moreover, SpoIVA is highly conserved in *Bacillus* and *Clostridium* species. Therefore, the aim of the present study is to investigate the mechanism of spore formation in *C. sporogenes* by performing a functional analysis of *spoIVA* encoding SpoIVA, a protein involved in the early development of the spore coat and cortex in *Bacillus subtilis*. Inactivation of *spoIVA* in *C. sporogenes* resulted in the loss of resistance of sporulating cells to lysozyme and heat treatments. Phase-contrast microscopy indicated that the inactivation of *spoIVA* caused the development of abnormal forespores and production of only a few immature spores. In the *spoIVA* mutant, abnormal swirl structures were detected in the mother cell using both phase-contrast and transmission electron microscopy. These swirls were stained with auramine O, pararosaniline hydrochloride, and 2-(4-aminophenyl)benzothiazole to examine the surface of mature spores of the wild-type strain. We found that the spore coat and exosporium proteins were misassembled and that they accumulated in the mother cells of the mutant. The results of this study indicate that SpoIVA is a spore morphogenetic protein, providing novel insights into spore morphogenesis in *C. sporogenes*.

## 1 Introduction

Gram-positive spore-forming bacteria belong to the orders Bacillales and Clostridiales. These species produce dormant endospores that can survive harsh conditions such as high heat, dry conditions, irradiation, and chemical exposure. Spore contamination leads to the spoilage of foods, beverages, and dairy products. The spores of *Bacillus cereus* can also cause food poisoning. Moreover, spores may contribute to the dissemination of pathogens such as *Clostridioides difficile* and *Bacillus anthracis*.

Genetic and morphological studies on spores were first conducted using the model bacterium *Bacillus subtilis* (Galperin et al., 2012). Advances in genome analysis have facilitated genetic comparisons among spore-forming Bacillota (formerly Firmicutes) species, revealing a conserved set of genes required for sporulation. The genes that encode sporulation-specific sigma factors, sigma E, F, G, and K, which play a crucial role in regulating gene expression during sporulation, are universally conserved in all spore-forming bacteria within the Bacillota phylum (Galperin et al., 2022). Some genes encoding proteins involved in asymmetric cell division, engulfment, cortex formation, spore coat formation, germination, and outgrowth are also conserved. Among these genes, *spoIVA*, which encodes the inner coat morphogenetic protein SpoIVA, is expressed under the control of sigma E in the mother cell (MC). In *B. subtilis* and *C. difficile*, spore coat assembly depends on SpoIVA (Roels et al., 1992; Driks, 1999; Catalano et al., 2001; Putnam et al., 2013). In *B. subtilis*, SpoIVA interacts with SpoVM, SpoIVA, and SpoVID to form the foundation layer of the spore coat. Additionally, it interacts with other morphogenetic proteins such as SafA and CotE (Driks, 1999). In *C. difficile*, SpoIVA and SipL form a complex that is essential for the correct assembly of the spore coat (Touchette et al., 2019). In a *B. subtilis spoIVA* mutant, both cortex and spore coat failed to fully develop, resulting in the formation of abnormal structures resembling swirls in the MC when observed using transmission electron microscopy (TEM) (Roels et al., 1992). Therefore, SpoIVA is essential for the formation of functionally resistant spores (Roels et al., 1992; Driks, 1999; Catalano et al., 2001). In *C. difficile, B. anthracis*, and *Bacillus thuringiensis*, *spoIVA* mutants exhibit defects in spore coat formation (Giorno et al., 2007; Putnam et al., 2013; Zhou et al., 2020), and abnormal structures have also been observed in the MC via TEM in *B. anthracis* and *C. difficile* (Giorno et al., 2007; Putnam et al., 2013). Among the four primary morphogenetic proteins involved in spore coat formation in *B. subtilis—*SpoIVA, SpoVID, SafA, and CotE—only SpoIVA is conserved in all endospore-forming organisms, suggesting a degree of diversity in spore coat assembly (Galperin et al., 2022).

*Clostridium* species are anaerobic Gram-positive spore-forming bacteria that are widely present in the intestines of animals and humans and in the environment. *Clostridium botulinum*, *Clostridium tetani*, *Clostridium perfringens*, and *C. difficile* are important pathogenic bacteria (Shen et al., 2019; Andryukov et al., 2021). Therefore, studies on the sporulation and germination of these *Clostridium* species are important. Moreover, facilities capable of treating *C. botulinum* and *C. tetani* infections are limited, owing to the high handling and biosafety level requirements associated with these highly pathogenic bacteria. *Clostridium sporogenes* are present in various places, including the soil and sediment of both marine and freshwater environments and preserved meat and dairy products (Brown et al., 2012). According to the 16S rRNA gene analysis, *C. sporogenes* NBRC 14293 exhibits physiological and genetic similarities to *C. botulinum* Group I (Williamson et al., 2016; Brunt et al., 2020). However, *C. sporogenes* NBRC 14293 is a non-pathogenic, putrefactive, spore-forming anaerobe used as a surrogate for *C. botulinum* (Brown et al., 2012; Brunt et al., 2020). *C. sporogenes* NBRC 14293 was initially isolated from cotton [Biological Resource Center, NITE (NBRC), Kisarazu, Chiba, Japan]. Spores produced by *C. sporogenes* can survive chemical, physical, and mechanical stressors, such as low levels of nutrients, the presence of oxygen, exposure to heat or high pressure, irradiation, and treatment with toxic chemicals (Henriques and Moran, 2007). The ability of these spores to persist in the environment poses a major challenge in food processing, negatively affecting effectiveness and safety, as spores can germinate and grow on food products, leading to spoilage and wastage.

Although morphological analysis of spores based on TEM observation has been performed (Mah et al., 2008; Brunt et al., 2015), the application of genetic recombination technology in *C. sporogenes* is uncommon, and only a few studies have focused on the spore-forming genes. We hypothesized that SpoIVA is involved in the dormancy and resistance of spores, similar to that in other spore-forming Bacillota species. In the present study, we constructed a spore formation-defective strain using ClosTron in *C. sporogenes* NBRC 14293, and characterized the function of SpoIVA. This study provides important insights into the prevention of anaerobic bacterial spore production and facilitates a comparative analysis of spore-forming genes among anaerobic spore-forming bacteria.

## 2 Materials and methods

### 2.1 Bacterial strains, plasmids, and media

*Clostridium sporogenes* and *Escherichia coli* strains used in this study are listed in Table 1. *C. sporogenes* NBRC 14293 obtained from the Biological Resource Center, NITE (NBRC) (Kisarazu, Chiba, Japan), was used in this study as the wild-type strain. *C. sporogenes* strains were cultured anaerobically in a Coy chamber (5% H_2_, 5% CO_2_, 90% N_2_) in Brain Heart Infusion medium (BHI; BD Biosciences, Franklin Lakes, NJ, USA) for genetic experiments or in a jar with Gifu Anaerobic Medium (GAM) (Nissui, Tokyo, Japan) in a deoxygenator (AnaeroPouch^®^ KENKI, Sugiyamagen, Tokyo, Japan). For solid media, agar was added at a final concentration of 17 g/L. When necessary, thiamphenicol (Tm, 15 μg/ml) or erythromycin (Erm, 2.5 μg/ml) was added to *C. sporogenes* culture. *E. coli* strains were cultured in Luria–Bertani (LB) broth. When indicated, ampicillin (Amp, 100 μg/ml) and chloramphenicol (Cm, 15 μg/ml) were added to the culture medium.

**TABLE 1:** Bacterial strains and plasmids used in this study.

Strain	Genotype	Origin
***Escherichia coli***		
NEB10	Δ*(ara-*leu*) 7697 araD139 fhuA*Δ*lacX74 galK16 galE15 e14-*ϕ*80*d*lacZ*Δ*M15 recA1 relA1 endA1 nupG rpsL* (Str^R^) *rph spoT1*Δ*(mrr-hsdRMS-mcrBC)*	BioLabs
HB101 (RP4)	*E*44 *aa*14 *galK*2 *lacY*1 Δ(*gpt*-*proA*) 62 *rpsL*20 (Str^R^)*xyl-5 mtl-1 recA*13 Δ(*mcrC*-*mrr*) *hsdS*_B_(r_B_^–^m_B_^–^) RP4 (Tra^+^ IncP Ap^R^ Km^R^ Tc^R^)	Laboratory stock
***Clostridium sporogenes***		
NBRC 14293	Wild-type	NBRC
CDIP1249	*spoIVA*:*erm*	This study
CDIP1488	*spoIVA*:*erm* pMTL83151-*spoIVA*	This study
**Plasmids**		
pGEM-T	Cloning vector for PCR, Ap^R^	Promega Corporation
pGEM-T *spoIVA*	pGEM-T containing the *spoIVA* gene region amplified by IMV1064 and RK3	This study
pMTL007-CE2	ClosTron vector for *C. sporogenes*	Heap et al., 2010
pDIA6780	pMTL007-CE2:*spoIVA* (160a)	This study
pMTL83151	*E. coli* -*C. sporogenes* shuttle vector	Heap et al., 2010
pDIA6974	pMTL83151-*spoIVA*	This study

### 2.2 Construction of a *spoIVA* mutant of *C. sporogenes*

As the genome sequence of *C. sporogenes* NBRC 14293 was not available, we first amplified and sequenced the *spoIVA* region. To achieve this, we used ClustalW2 to multi-align *spoIVA* sequence using the complete genome of several *C. sporogenes* strains: DSM 795, NCIMB 10696, ATCC 3584, and PA 3679. We then designed oligonucleotides IMV1064 and RK3 to amplify a 1.5-kb DNA fragment using the DNA of *C. sporogenes* NBRC 14293 as a template (Supplementary Figure 1A). The resulting polymerase chain reaction (PCR) fragment was inserted into the pGEM-T vector (Promega, Madison, WI, USA) using TA cloning, producing pGEM-T *spoIVA*. Three independent clones were sequenced using primers T7 and SP6 to determine the sequence of *spoIVA*. We subsequently inactivated *spoIVA* in *C. sporogenes* NBRC 14293 using the ClosTron mutagenesis system (Heap et al., 2007, 2010). For retargeting the Group II Ll.LtrB intron of pMTL007-CE2 to *spoIVA*, primers (Supplementary Table 1) were designed using an online tool provided by the University of Nottingham (Heap et al., 2007). PCR primer sets were used with the EBS universal primer and intron template DNA. The aim was to generate a 353-base pair product using overlap extension PCR that would facilitate intron retargeting. The PCR product was cloned into *Hin*dIII and *Bsr*GI restriction sites of pMTL007-CE2 to produce pDIA6780 (pMTL007-CE2 Csp-*spoIVA-*160a) (Supplementary Figure 1B). DNA sequencing was performed to confirm the plasmid constructs using the pMTL007-specific primer pMTL007-R. The plasmid pDIA6780 was subsequently transferred into *C. sporogenes* NBRC 14293 via conjugation with *E. coli* strain HB101 (RP4). Thiamphenicol-resistant *C. sporogenes* clones were selected and plated on BHI plates supplemented with Tm (15 μg/ml), and then on BHI agar containing erythromycin (2.5 μg/ml) to confirm the integration of Group II intron into *spoIVA*. PCR was performed using two primer pairs: one flanking the integration site in *spoIVA* (RK1-RK8) and the second included a primer in *spoIVA* (RK8), and another in the intron (EBSu) (Supplementary Table 1 and Supplementary Figure 1B). The PCR fragments were sequenced to confirm that the intron was inserted into *spoIVA* between nucleotides 160 and 161 in an antisense orientation.

The shuttle vector pMTL83151 (Heap et al., 2009) was used to complement *spoIVA* mutants (Heap et al., 2009). A DNA fragment containing *spoIVA* and its promoter region (−557 bp from the translational start site to +72 bp after the stop codon) was amplified via PCR using the primers IMV1132 and IMV1133. The PCR fragment was cloned into *Bam*HI and *Xho*I restriction sites of the shuttle vector pMTL83151 to yield pDIA6974. Plasmid pDIA6974 was transferred into a *spoIVA* mutant of *C. sporogenes* via conjugation with HB101 (RP4). Tm-resistant *C. sporogenes* clones were selected for further analysis.

### 2.3 Heat and lysozyme resistance of spores

*Clostridium sporogenes* cells were cultured in GAM at 37°C for 24 and 48 h, and spore resistance was assayed. The cultures were either heated at 80°C for 20 min or treated with lysozyme (250 μg/mL final concentration) at 37°C for 10 min as previously described (Kuwana et al., 2003). The samples were them serially diluted in distilled water. Sample volumes of the appropriate dilutions were determined and spread on GAM agar plates and incubated at 37°C for 24 h. The number of colony-forming units (CFUs) and spores per milliliter for each strain was initially determined by counting the colonies. The percentage of sporulation was determined as the ratio of the number of spores per milliliter to the total number of CFUs per milliliter (×100). The results are presented as mean of at least three independent trials.

### 2.4 Phase-contrast and fluorescent microscopy

After 24 or 48 h of growth in GAM agar at 37°C, *C. sporogenes* cells were stained using acridine orange 10-nonyl bromide (nonyl acridine orange, NAO) (final concentration 0.01 mg/ml) (Santa Cruz Biotechnology, Dallas, TX, USA), Hoechst 33342 (final concentration 0.01 mg/ml) (Thermo Fisher Scientific, Waltham, MA, USA), 2-(4-aminophenyl)benzothiazole (APBT) (final concentration 0.01 mg/ml) (Tokyo Chemical Industry, Tokyo, Japan), auramine O (final concentration 0.01 mg/ml) (Tokyo Chemical Industry), or pararosaniline hydrochloride (final concentration 0.01 mg/ml) (Nacalai Tesque, Inc., Kyoto, Japan). The cells were incubated with the products in 10 mM Tris–HCl (pH 7.6) for 10 min at 25°C, and the samples were transferred onto microscope slides as previously described (Kuwana et al., 2022, 2023). Pararosaniline hydrochloride is a fluorescent probe used for the spectrofluorometric determination of ondansetron, an antiemetic drug (Essawy and Ali, 2018).

Phase-contrast and fluorescence images of *C. sporogenes* cells were obtained using an Olympus BX51 phase-contrast microscope with additional fluorescence tools and mirror cube units (Olympus, Tokyo, Japan). The green fluorescence of auramine O and NAO was detected using a mirror cube unit (U-MGFPHQ). The red fluorescence of pararosaniline hydrochloride was detected using a mirror cube unit (U-MWG2). The blue fluorescence of APBT and Hoechst 33342 was detected using a mirror cube unit (UMNUA2). A UPlanApo 100× oil Iris Ph3 objective lens and a U-TV1X-2 camera adapter were used (Olympus). Images were captured using an ORCA-SPARK digital CMOS camera C11440-36U (Hamamatsu Photonics Inc., Shizuoka, Japan) and analyzed using the cellSens imaging software (Olympus). The exposure time for image capture of each fluorescence dye was 0.4–4.0 s. The captured images were processed using cellSens imaging software (Olympus) for minor adjustments in brightness, contrast, and color balance, and the creation of merged images. Images for each strain were scaled to the same intensity range.

### 2.5 Transmission electron microscopy (TEM)

*Clostridium sporogenes* strains were cultured in GAM at 37°C for 24 h. Sporulating cells were fixed with 2.5% glutaraldehyde and 2% OsO_4_, and subsequently embedded in Quetol 653. Thin sections of spores and sporulating cells were stained with 3% (w/v) uranyl acetate and observed under a JEM-1200EX electron microscope at 80 kV (Kuwana et al., 2003).

### 2.6 Statistical analyses

To determine statistical significance, a two-way analysis of variance (ANOVA), followed by Turkey’s multiple comparisons test, was used to compare the control group with the heat or lysozyme treatment group. To determine the significance of spore resistance, a one-way ANOVA was performed, followed by Tukey’s multiple comparisons test. Results with *P* ≤ 0.05 were considered statistically significant, and all statistical analyses were performed using Microsoft Excel.

## 3 Results

### 3.1 *spoIVA* disruption affects the resistance of *C. sporogenes* spores

**FIGURE 1:**
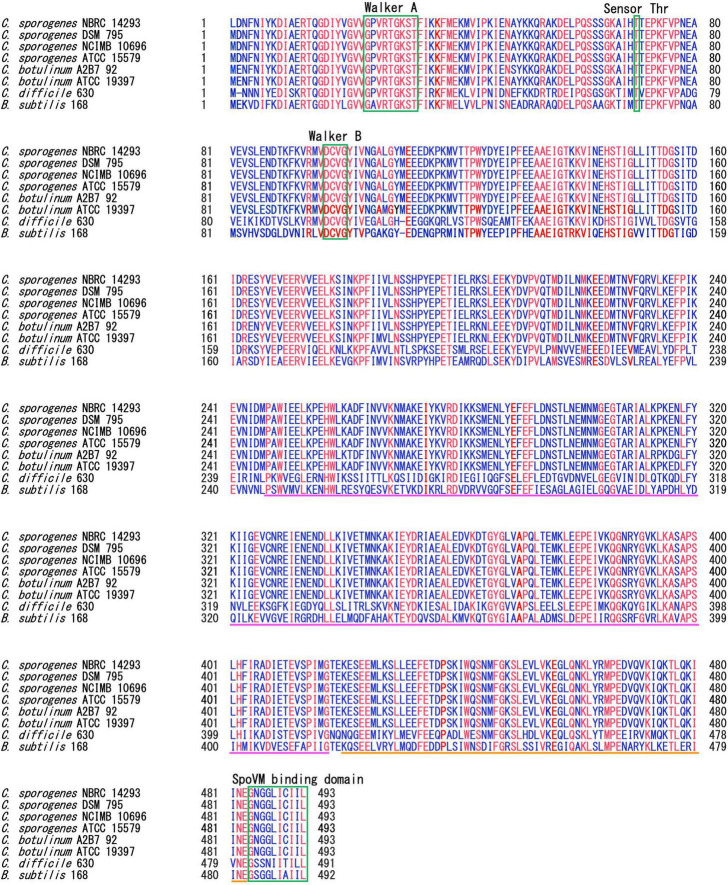
Multiple alignments of SpoIVA amino acid sequences. Multiple alignments of SpoIVA sequences of *Clostridium sporogenes*, *Clostridium botulinum*, *Clostridium difficile*, and *Bacillus subtilis*. The Walker A motif is required for ATP binding, whereas the threonine and Walker B motifs are required for ATP hydrolysis, as demonstrated in *B. subtilis* SpoIVA (Castaing et al., 2014; Benito de la Puebla et al., 2020). The C-terminal region of SpoIVA, boxed in green, has been implicated in binding to SpoVM. The pink line shows the central domain, which is required for conformational changes induced by ATP hydrolysis in *B. subtilis* SpoIVA. The orange line represents the globular C-terminal domain. Blue text indicate highly conserved amino acids; red text indicates completely conserved amino acids among these strains.

SpoIVA is highly conserved among spore-forming Bacillota species (Galperin et al., 2012, 2022). SpoIVA of *C. sporogenes* NBRC 14293 shares 99.8%, 99.8%, 99.6%, 97.5%, 96.55%, 56.0%, and 52.7% identity with the protein of *C. sporogenes* DSM 795, NCIMB 10696, ATCC 15579, *C. botulinum* A2B7 92, ATCC 19397, *C. difficile* 630, and *B. subtilis* 168, respectively. We also performed alignment of SpoIVA of *C. sporogenes* DSM 795, NCIMB 10696, ATCC 15579, *C. botulinum* A2B7 92, ATCC 19397, *C. difficile* 630, and *B. subtilis* 168 (Figure 1).

**FIGURE 2:**
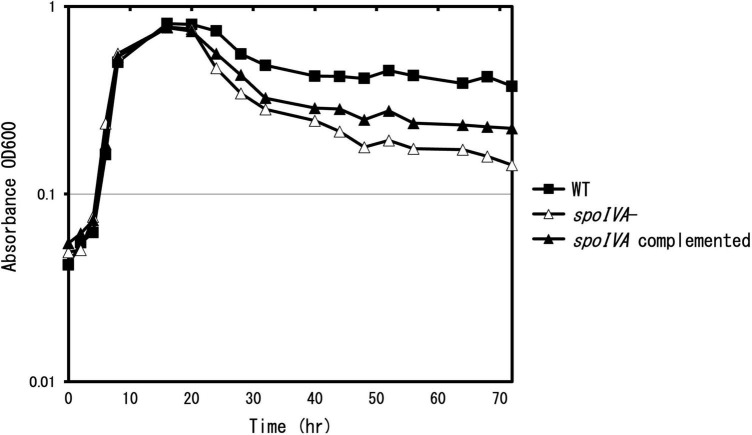
Growth of *Clostridium sporogenes* strains. *C. sporogenes* NBRC 14293 (closed square), the *spoIVA* mutant (open triangle), and the complemented strain (closed triangle) cells were grown in GAM at 37°C. Growth curves were obtained by measuring optical density at 600 nm (OD_600__nm_). Data are presented as mean ± SD calculated from at least three independent experiments.

To determine the role of SpoIVA in the sporulation and coat assembly of *C. sporogenes*, we generated a *spoIVA*:*erm* mutant using the ClosTron system in *C. sporogenes* NBRC 14293 strain. We complemented the mutant strain with the plasmid pMTL83151 carrying *spoIVA* expressed under the control of its promoter region. The cells were grown in GAM at 37°C. After inoculation, OD_600__nm_ was measured regularly (Figure 2). During the exponential growth (0–16 h) and early stationary phases (16–24 h), the *C. sporogenes spoIVA* mutant and parental strains grew similarly in liquid GAM and on GAM agar. However, during the late stationary phase (24–72 h), the OD_600__nm_ of the *spoIVA:erm* mutant decreased faster than that of the wild-type cells. The growth of the *spoIVA*-complemented strain partially recovered during the late stationary phase. A comparison of the growth curves indicated that inactivation of *spoIVA* affected the late stationary phase.

**FIGURE 3:**
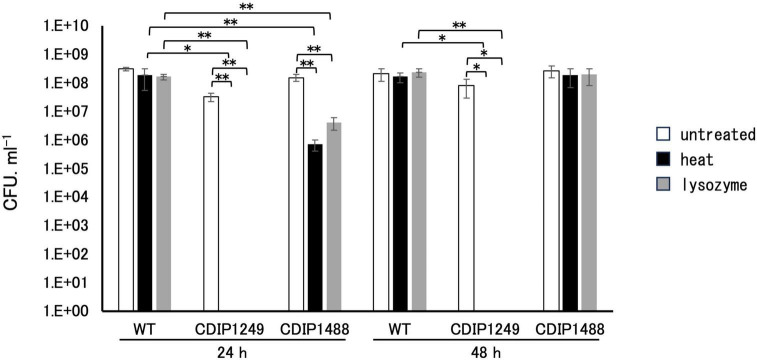
Resistance of *Clostridium sporogenes* spores. Spores were spread on GAM agar after heating at 80°C for 20 min or following incubation with lysozyme (250 μg/mL final concentration) at 37°C for 10 min. The viability rate was determined at 24 and 48 h by counting the colonies in the presence or absence of treatment. White, black, and gray bars indicate untreated, heat-treated samples, and lysozyme-treated samples, respectively. Data are presented as mean ± SD calculated from at least three independent experiments. Asterisks indicate significant differences, which were determined via two-way ANOVA followed by Tukey’s multiple comparison test to compare between conditions. *P < 0.05, **P < 0.01.

To confirm the crucial role of SpoIVA in sporulation, we compared the sporulation efficiency of NBRC 14293 strain, the *spoIVA* mutant, and the complemented strain after culture at 37°C in GAM. After 24 h, 60% and 53% of the total cells corresponded to spores resistant to heat and lysozyme of the wild-type strain (Figure 3), and the efficiency of sporulation reached almost 100% after 48 h. The inactivation of *spoIVA* abolished the formation of spores resistant to heat or lysozyme at 24 and 48 h (Figure 3). The complementation of the *spoIVA*:*erm* mutant with a plasmid carrying *spoIVA* partially restored the formation of spores resistant to heat or lysozyme after 24 h. However, the resistance of spores to heat and lysozyme after 48 h was similar to that of the wild-type strain. A delay in sporulation was observed for the complemented strain compared to the wild-type strain, and this delay might be attributed to the presence of a plasmid or the overexpression of *spoIVA* in this strain. These results indicate that SpoIVA is necessary for sporulation and/or resistant spore production.

### 3.2 Designation of sporulation stages in *C. sporogenes* NBRC 14293

**FIGURE 4:**
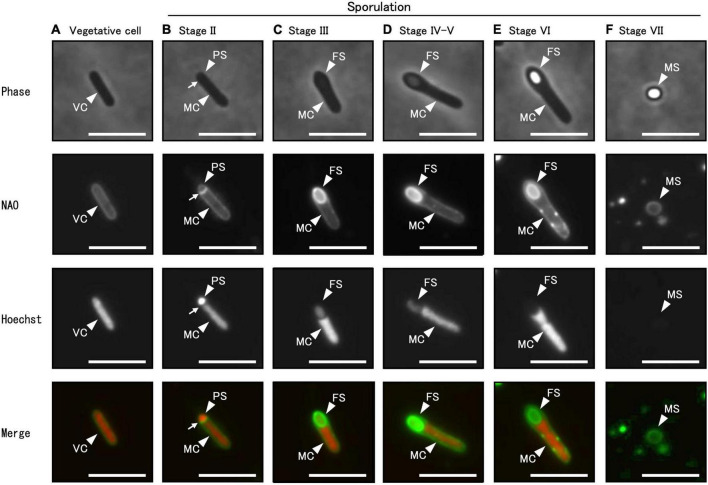
Different sporulation stages of *C. sporogenes* NBRC 14293. *C. sporogenes* cells were grown on GAM agar at 37°C for 24 h **(A–E)** or 48 h **(F)**. Aliquots of cell suspensions were analyzed either via phase-contrast microscopy (first panel) or using fluorescence microscopy after staining with NAO (second panel) and Hoechst 33342 (third panel). Merged images are shown (fourth panel). Hoechst 33342 and NAO were used to detect chromosomal DNA and cell membrane, respectively. We identified different sporulation stages of *C. sporogenes* NBRC 14293: vegetative cells (VC), mother cells (MC), prespores (PS), forespores (FS), and mature spores (MS). Arrows indicate asymmetric cell division sites. Scale bars represent 5 μm.

Using phase-contrast and fluorescent microscopy, we analyzed the successive morphological changes in *C. sporogenes* NBRC 14293 strain as it transitioned from vegetative cells to spores in GAM at 37°C. Samples included vegetative cells, cells at all sporulation stages, and mature spores (Figure 4). Using a phase-contrast microscope (Figure 4, upper panel), we observed vegetative cells (Figure 4A), mother cell (MC) and prespores (PS) (Figure 4B), phase-gray forespores (FS) (Figures 4C, D), and phase-bright spores (Figure 4E) in the MC. To distinguish the prespore, forespore, and MC from vegetative cells before coat and cortex development, we used fluorescent dyes and fluorescence microscopy. Cell membranes and DNA were stained with NAO and Hoechst 33342, respectively. We observed and compared the different sporulation stages of *C. sporogenes* NBRC 14293 with those identified in other endospore-forming organisms, such as *B. subtilis* 168, *B. cereus* ATCC 14579, and *C. botulinum* type B strain 111 (Nicholson and Setlow, 1990; Errington, 1993; Hosomi et al., 2015; Kuwana et al., 2022). Rod-shaped cells, with DNA enclosed in a cytoplasmic membrane without asymmetric division, indicated the progression from vegetative growth to stage II sporulation (Figure 4A). After asymmetric cell division, small and large cells separated by the membrane corresponded to PS and MC, respectively (Figure 4B, Stage II). Hoechst 33342 fluorescence was detected in both PS (or FS) and MC during stages II and III (Figures 4B, C). Oval-shaped forespores were visible within the rod-shaped mother cells displaying NAO fluorescence at stage III (Figure 4C). The PS completely engulfed by the MC became FS. Hoechst 33342 fluorescence was also detected in FS (Figure 4C). Phase-gray FS were observed using phase-contrast microscopy (Figure 4D). The impermeability of Hoechst 33342 gradually increased from stage IV. We observed less refractive FS compared to mature spores at stage IV sporulation (Figure 4D) and fully refractive, phase-bright FS within the MC at stage VI sporulation (Figure 4E). We detected NAO fluorescence in the MC membrane and the outer layer of the FS from stages III to VI, whereas the fluorescence of Hoechst 33342 was not detected in the FS from stages VI to VII (Figures 4E, F). Finally, *C. sporogenes* NBRC 14293 produced an oval-type endospore at the terminus of the bacterium. The release of spores by MC lysis corresponded to stage VII (Figure 4F). Mature free spores were slightly stained with NAO but not with Hoechst 33342 (Figure 4F).

### 3.3 Abnormal structures formed in the *spoIVA*-mutant sporulating cells

**FIGURE 5:**
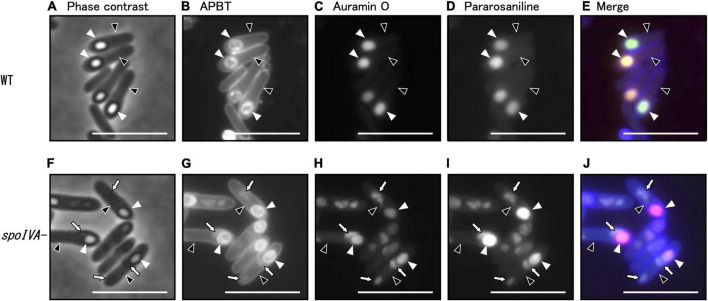
Analysis of morphology of the *spoIVA* mutant using fluorescence microscopy. *C. sporogenes* NBRC 14293 **(A–E)** and *spoIVA* mutant **(F–J)** cells were grown on GAM agar at 37°C for 24 h. The cells were stained with a combination of APBT, auramine O, and pararosaniline hydrochloride. The cells were analyzed using phase-contrast microscopy and fluorescence microscopy with a mixture of fluorescent dyes. Phase-contrast **(A,F)**, APBT fluorescent **(B,G)**, auramine O fluorescent **(C,H)**, pararosaniline hydrochloride fluorescent **(D,I)**, and merged images **(E,J)** are shown. Black arrowheads indicate mother cells, white arrowheads indicate forespores, and arrows indicate abnormal structures in the mother cells. Scale bars represent 5 μm.

Based on the classification of cell morphology shown in Figure 4, we subsequently analyzed sporulation in the *spoIVA*:*erm* mutant (Figure 5). We observed the *C. sporogenes* parental strain and *spoIVA*:*erm* mutant cultured in GAM at 37°C for 24 h using phase-contrast microscopy. We analyzed cells stained with a combination of APBT, auramine O, and pararosaniline hydrochloride using fluorescence microscopy (Figure 5). The synthetic basic dye pararosaniline hydrochloride binds to acidic structures, especially nucleic acids and proteins, and intensely stains them red. It is commonly used for microbial staining and as an industrial agent in a wide variety of commercial products including paper, textiles, cosmetics, and paint (Huynh and Wu, 2023; Shetty et al., 2023). APBT stains the cell membrane and/or cell wall in *B. subtilis* cells, auramine O stains forespore and mature spore (Kuwana et al., 2023). Phase-contrast microscopy revealed phase-bright FS in the MC of both wild-type strain and *spoIVA*:*erm* mutant during sporulation (Supplementary Figure 2; green arrows). However, the *spoIVA*:*erm* mutant exhibited several phase-gray FS and abnormal structures within the MC. Moreover, we observed phase-bright refractive-free spores in the wild-type strain, but not in the *spoIVA*:*erm* mutant (Supplementary Figure 2). We observed free phase-gray spores in *spoIVA*:*erm* mutant cells (Supplementary Figure 2, orange arrows).

We counted wild strain and *spoIVA*:*erm* mutant cells in different sporulation stages based on phase-contrast and fluorescence microscopy images of cells stained with APBT (Table 2 and Supplementary Figure 2). We classified the cells into five sporulation stages: vegetative cells, sporulation stages II to III, sporulation stages IV to V, sporulation stage VI, and sporulation stage VII. We found that the progression of sporulation in *spoIVA*:*erm* mutant cells was similar to that of the wild-type strain (Table 2). In the vegetative cells and sporulation stages II–III, we observed no distinguishable abnormal structures in the *spoIVA*:*erm* mutant. However, in sporulation stage IV and later, we observed abnormal structures of only the *spoIVA*:*erm* mutant (Figure 5 and Supplementary Figure 2). Moreover, we observed irregular FS shapes and higher refractive indices in MCs of the mutant (Figure 5F, arrows).

**TABLE 2:** Proportion of sporulation stage cells in *Clostridium sporogenes* NBRC 14293 and *spoIVA* mutant strains.

	NBRC 14293		CDIP1249 (*spoIVA*:*erm*)	
	Number of cells	Percentage (%)	Number of cells	Percentage (%)
Vegetative cells	56	10.9	51	12.9
Stages II–III	50	9.7	47	11.9
Stages IV–V	70	13.6	235	59.6
Stage VI	319	62.1	53	13.5
Stage VII	19	3.7	8	2.0

Cells were cultured on GAM for 24 h. The cells were stained with APBT, and then subjected to phase-contrast and fluorescence microscopy. From the results of microscopic observations (Figure 5 and Supplementary Figure 2), the number of cells in each sporulation stage and their percentage were determined. Cell counts were analyzed from microscopic images obtained by three independent experiments. Stage IV–V and stage VII showed a significant difference in the number of cells between the two backgrounds (the *P*-value of stage IV–V and stage VII is less than 0.05, respectively).

In *B. subtilis*, we have previously demonstrated that auramine O effectively stained the FS from stage VI and mature spores, whereas APBT stained the membranes of vegetative cells and MCs and the periphery of the FS and mature spores (Kuwana et al., 2022). In the present study, we stained the wild-type and *spoIVA*:*erm* mutant strains of *C. sporogenes* during sporulation and observed APBT fluorescence in the cellular membrane and FS of the wild-type and *spoIVA*:*erm* mutant cells (Figures 5B, G). In wild-type cells, we detected fluorescence associated with APBT both around the FS and within the MC (Figure 5B). In the *spoIVA*:*erm* mutants, we detected APBT fluorescence surrounding the FS and some abnormal structures within the MC (Figure 5G, arrows). Both auramine O and pararosaniline hydrochloride effectively stained the FS in both the wild-type and mutant strains (Figures 5C, D, H, I). However, the staining also revealed abnormal structures within the sporulating cells of the *spoIVA*:*erm* mutant (Figures 5H, I, arrows). APBT, auramine O, and pararosaniline hydrochloride can stain the mislocalized spore coat in the mother cell of the *spoIVA* mutant. These findings confirm the presence of defects in the sporulating cells of the *spoIVA* mutant.

### 3.4 Morphological observation of *C. sporogenes* NBRC 14293 and *spoIVA* mutant cells using TEM

**FIGURE 6:**
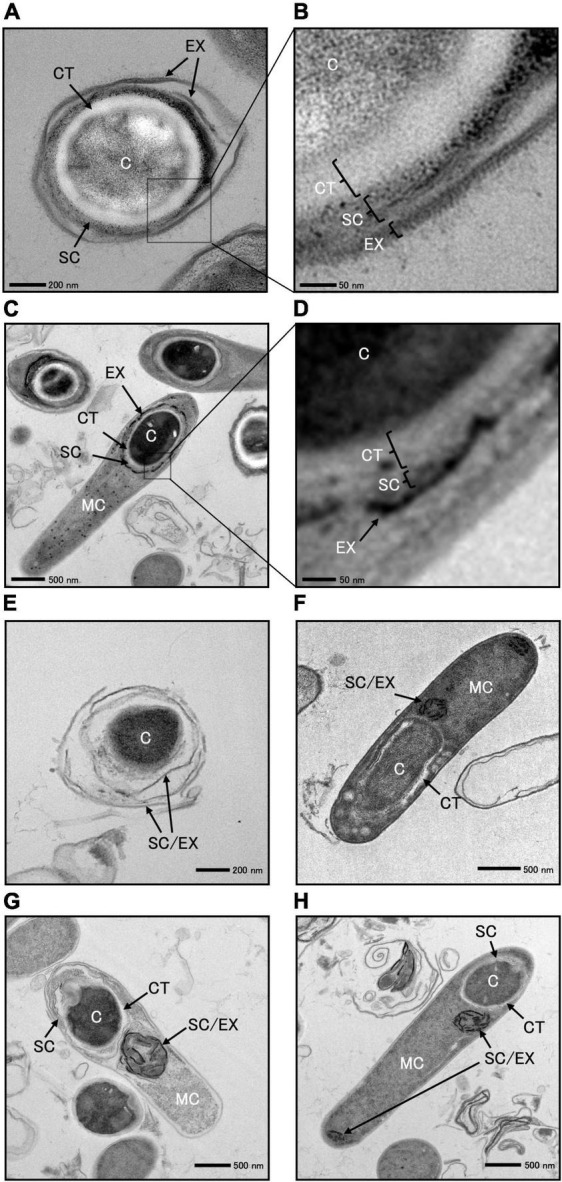
Transmission electron microscopy of *Clostridium sporogenes* NBRC 14293 and *spoIVA* mutant sporulating cells and spores. *C. sporogenes* NBRC 14293 strain was grown on GAM at 37°C for 24 h and analyzed using transmission electron microscopy. The wild type mature spores **(A,B)** and sporulating cells **(C,D)** are shown. Panels **(B,D)** show enlarged images of panels **(A,C)**, respectively. The exosporium (EX), spore coat (SC), cortex (CT), and core (C) were observed in the spore. Mother cells (MC), exosporium (EX), spore coat (SC), cortex (CT), and core of the forespore (C) were observed in sporulating cells. The *spoIVA* mutant immature spores **(E)** and sporulating cells **(F–H)** are shown. The exosporium and spore coats were indistinguishable. Abnormal spore coats and/or exosporium structures (SC/EX) were observed in the *spoIVA* mutant immature spores and sporulating cells. The sizes indicated with scale bars are shown in respective panels.

We cultured *C. sporogenes* NBRC 14293 strain and the *spoIVA*:*erm* mutant in GAM at 37°C for 24 h and analyzed the ultrastructure of wild-type and *spoIVA*:*erm* sporulating cells and spores using TEM (Figure 6 and Supplementary Figure 3). TEM images revealed the presence of four major structures in the mature spores of *C. sporogenes* NBRC 14293: the core, cortex, spore coat, and exosporium (Figure 6). The spore coat and exosporium exhibited a high electron density, appearing dark, whereas the cortex, characterized by low electron density, appeared bright. In the sporulating cells of *C*. *sporogenes* NBRC 14293, the dehydrated core of the FS appeared dark, whereas the cortex on the outside of the core was brightly visible (Figures 6C, D). The FS were surrounded by the coat and/or the exosporium materials, detected as high electron-dense structures. In mature spores, the core was dehydrated and appeared bright (Figures 6A, B), and the spore coat layers were gray between the exosporium layer and cortex. The cortex layer appeared thicker than the coat layer. Finally, the exosporium was attached to the outermost portion of the spore coat (Figures 6A, B).

We observed a few spores in the *C. sporogenes spoIVA*:*erm* mutant (Figure 6E and Supplementary Figure 2). The core of these spores remained dark, indicating no dehydration. The cortex layer was not detected, whereas structures resembling spore coats and/or exosporium were observed covering the spores (Figure 6E). We also observed immature and heteromorphic spores enclosed within abnormal structures, making it difficult to identify the cortex, spore coat, and exosporium (Figures 6F–H). Within the FS of sporulating cells, a low electron-dense cortex layer surrounded the core. In the *spoIVA*:*erm* mutant, this cortex layer appeared more irregular in thickness and thinner than that in the wild-type strain. The high electron-dense layer observed around the FS in the wild-type strain was not observed in sporulating cells of the *spoIVA* mutant (Figures 6F–H). Finally, abnormal structures resembling swirls were observed within the MC (Figures 6F–H). An incomplete thin-layered structure, possibly the spore coat, was observed around the undeveloped cortex of the FS (Figures 6F–H). These results highlight the importance of SpoIVA for normal development of the cortex, spore coat, and exosporium in *C. sporogenes*.

## 4 Discussion

A limited number of studies have been performed using mutant strains constructed via genetic recombination technology in *C. sporogenes*. In the present study, we generated, using the ClosTron system, a mutant with inactivated *spoIVA*, which encodes SpoIVA—a protein conserved in spore-forming bacteria and is involved in the morphogenesis of the spore coat. We found that the function of SpoIVA is essential for spore formation akin to its role in *B. subtilis*, *B. thuringiensis*, and *C. difficile* (Catalano et al., 2001; Putnam et al., 2013; Zhou et al., 2020).

In the present study, GAM supported the growth of *C. sporogenes* NBRC 14293 in an anaerobic jar incubated at 37°C, reaching the stationary phase 16 h after inoculation. The 24-h cultures contained a mixture of vegetative and sporulating cells at different stages, from asymmetric division to mature free spores (Table 2). We also detected approximately 10^8^ spores per milliliter that were resistant to heat and lysozyme. As observed in other spore-forming bacteria (Roels et al., 1992), *spoIVA* inactivation did not affect the exponential growth phase. There was also no significant difference in the process of sporulation between the wild-type strain and *spoIVA*:*erm* mutant during early sporulation stages. The *spoIVA* inactivation exhibited that the majority of sporulating cells were phase gray spores (stage IV–V) during the late sporulation stages. However, a reduced number of sporulating cells or spores without any defect was detected in the *spoIVA* mutant, potentially explaining the reduction in OD_600__nm_ observed during the late stationary phase for the *spoIVA* mutant. This reduction is likely associated with increased lysis. Moreover, as observed in *B. subtilis* and *C. difficile* (Stevens et al., 1992; Tan et al., 2015; Benito de la Puebla et al., 2020), the *spoIVA* mutant of *C. sporogenes* failed to acquire heat or lysozyme resistance in our study. We found a small amount of the free phase-gray spores in the *spoIVA* mutant, which is a unique phenotype to the *C. sporogenes spoIVA* mutant. Moreover, the TEM analysis revealed no cortex structure, and the coat and exosporium exhibited abnormal structures in the *spoIVA* mutant strain.

Complementation of *spoIVA* using a plasmid may have slightly affected the heat and lysozyme resistance of the spores at 24 h after culture. However, at 48-h of culture, the resistance was similar to that of the wild-type strain. Indeed, a previous study in *C. perfringens* has demonstrated the failure of plasmid-complemented mutants to regain wild type sporulation levels (Li et al., 2011). Moreover, the use of multicopy plasmids can fail to restore a wild type phenotype in *Clostridium* species (Li et al., 2011; Ng et al., 2013; Brunt et al., 2014, 2016, 2018; Meaney et al., 2015). In addition, it has been shown that the polymerization of SpoIVA occurs *in vitro* at a constant threshold (Castaing et al., 2014). The complementation approach using a multicopy plasmid may lead to an overproduction as observed for SpoIVA in *C. difficile* (Putnam et al., 2013). SpoIVA overproduction might allow to reach a critical concentration high enough to allow spontaneous polymerization leading only to a partial complementation.

While the basic morphological changes that occur during spore morphogenesis are conserved between *Clostridium* and *Bacillus* species, the underlying genetic orchestration and regulation differ (Al-Hinai et al., 2015). In the present study, we defined the sporulation stage based on the morphological changes in cells as observed using phase-contrast and fluorescence microscopy in *C. sporogenes* NBRC 14293. These morphological changes are similar to those described for *B. subtilis*, *B. cereus*, and *C. botulinum* (Errington, 1993; Al-Hinai et al., 2015; Hosomi et al., 2015; Kuwana et al., 2022). Using TEM, we also analyzed the structure of *C. sporogenes* NBRC 14293 mature spores, revealing the presence of a core, cortex, spore coat, and exosporium. The TEM spore images of *C. sporogenes* NBRC 14293 resembled those of other *C. sporogenes* strains PA 3679, ATCC 15579, and DSM 795, and *C. botulinum* strains 78A, ATCC 3502, ATCC 19397, and UN1/10-7B. These *C. botulinum* strains are classified under Group I (Stevenson and Vaughn, 1972; Mah et al., 2008; Brunt et al., 2015; Poehlein et al., 2015; Portinha et al., 2022). Unlike the mature spores of *B. subtilis* 168 and *C. difficile* R20291 (Driks, 1999; Baloh et al., 2022), there were no lamellar structures. Moreover, we could not distinguish between the inner and outer coats of the mature spores of *C. sporogenes* NBRC 14293, as observed for *C. sporogenes* ATCC 15579 (Brunt et al., 2015). Bacterial spores can be classified into two categories: those containing a distinct exosporium and those lacking this structure (Stewart, 2015; Driks and Eichenberger, 2016). In the present study, surrounding the periphery of the forespore, dark layers with high-electron density were observed, likely corresponding to an exosporium and strongly suggesting that *C. sporogenes* NBRC 14293 has a distinct exosporium structure, similar to those of the spores of *C. botulinum* Group I strains and *C. sporogenes* ATCC 15579 (Stevenson and Vaughn, 1972; Brunt et al., 2015; Portinha et al., 2022). Morphologically, *C. sporogenes* spores are considerably different from *C. difficile* spores; the exosporium layer of *C. difficile* spores is bumpy with electron-dense and hair-like projections on the surface of *C. difficile* CD196 and R20291 spores (Rabi et al., 2017; Paredes-Sabja et al., 2022). However, we did not identify such structures on the surface of *C. sporogenes* NBRC 14293 spores.

SpoIVA is a morphogenetic protein produced in the MC under the control of sigma E in *B. subtilis* and *C. difficile* and is essential for the early stages of spore coat assembly and cortex production, leading to the formation of resistant spores (Catalano et al., 2001; Putnam et al., 2013). However, the synthesis of other spore coat proteins persists, leading to the formation of abnormal aggregates in the MC of *spoIVA* mutants of *B. subtilis* and *C. difficile* 630 (Roels et al., 1992; Putnam et al., 2013). In *C. sporogenes* NBRC 14293, we demonstrated that SpoIVA is involved in spore formation and that the *spoIVA* mutant is more sensitive to heat and lysozyme treatments than the parental strain. We also detected abnormal structures in the MC of the *spoIVA* mutant. These abnormal structures, which were weakly stained by the membrane marker APBT, are not cytoplasmic membranes, but rather hydrophobic materials present in the cytoplasm of the MC. Their positive staining with both auramine O and pararosaniline also suggests the presence of acidic substances in the abnormal structures. The TEM analysis of *C. sporogenes* NBRC 14293 *spoIVA* mutants confirmed the presence of swirls in MC, as observed in *B. subtilis* and *C. difficile* (Roels et al., 1992; Putnam et al., 2013). In free mature spores of the wild-type *C. sporogenes* strain, the exosporium and spore coat appeared as highly electron-dense layers, yet they could be clearly distinguished. In the forespore, the exosporium was attached to the outer side of the spore coat. In the *spoIVA* mutant, the highly electron-dense thin layers lapping free spores did not distinguish the spore coat from the exosporium. In the MC of the *spoIVA* mutant, the highly electron-dense layer swirls were composed of a misassembled spore coat and/or exosporium materials, as previously observed in *B. subtilis*, *B. anthracis*, and *C. difficile* mutants (Roels et al., 1992; Giorno et al., 2007; Putnam et al., 2013; Benito de la Puebla et al., 2020). Furthermore, the core of a few *spoIVA* spores remained dark owing to the lack of dehydration and underdevelopment of the cortex layer. The *spoIVA* mutant of *B. subtilis* lacks a cortex (Roels et al., 1992), whereas the *spoIVA* mutant of *C. difficile* produces a cortex even if some abnormalities in cortex thickness are observed (Putnam et al., 2013). In the present study, the cortex was observed in the forespore, but not in the immature free spores of *C. sporogenes spoIVA* mutant. The few immature *spoIVA* mutant spores detected were sensitive to heat and lysozyme. Notably, the presence of free spores in *spoIVA* mutants has not yet been reported in other species (Zhou et al., 2020). We propose that the drastic reduction in the cortex layer may be due to abnormal cortex digestion by degradative enzymes; however, this does not cause complete lysis of all free spores in the *C. sporogenes spoIVA* mutant. SpoIVA is required for the complete development of the cortex, assembly of the spore coat, and exosporium in *C. sporogenes*. Under the phase contrast microscopy, forespores with phase-dark outlines are present in the wild-type and the *spoIVA* mutant cells as observed in the wild-type, the *spoIVA* mutant, and the *sipL* mutant of *C. difficil*e (Touchette et al., 2021). Phase-dark outlines around forespores are typically observed when forespores become phase-bright due to the dehydration of the forespore cytosol as it matures, which occurs when the thick cortex layer is synthesized (Popham and Bernhards, 2015). We detected some phase-bright refractive forespore in the *C. sporogenes spoIVA* mutant sporulating cells corresponding to stage VI. Only phase-dark and phase-gray forespores blocked at stage IV are produced by the *spoIVA* mutant of *C. difficile* (Putnam et al., 2013; Benito de la Puebla et al., 2020). McKenney and Eichenberger (2012) reported that phase-dark forespores can be distinguished between sporulation stages IV and V, and phase-bright forespores can be distinguished in stage VI in *B. subtilis* using phase-contrast microscopy. The phenotype of *C. sporogenes spoIVA* mutant strain may be consistent with that of sporulation stage VI instead of stage IV, as observed in other endospore-forming Bacillota species. While the primary structure and function of SpoIVA in *C. sporogenes* are also conserved among *Bacilli* and *Clostridia* species, events following sporulation stage IV, including the autolytic process, appear to be different in *C. sporogenes*.

In the present study, the SpoIVA protein multi-alignment indicated the presence of conserved motifs. The ATPase domain is highly conserved in SpoIVA among *Bacillus* and *Clostridium* species (Castaing et al., 2014). ATPase activity drives SpoIVA polymerization around the forespore. This may also be the case for the *C. sporogenes* SpoIVA protein, which contains the Walker A motif and threonine necessary for ATPase activity (Benito de la Puebla et al., 2020). SpoIVA from *B. subtilis* and *C. difficile* also binds to the small amphipathic SpoVM protein via their C-terminal domain. Inactivation of *spoVM* leads to major defects in coat assembly and cortex formation in *B. subtilis* (Levin et al., 1993), whereas SpoVM appears to play only a small role in coat assembly in *C. difficile* (Ribis et al., 2017). This study identified SpoVM in the genome of *C. sporogenes* and *C. botulinum*, and a SpoVM-binding domain was identified in the SpoIVA protein of *C. sporogenes* and *C. botulinum*. SpoIVA is highly conserved among the four *C. sporogenes* strains, ATCC 15579, DSM 795, NCIMB 10690, and NBRC 14293. The SpoVM-binding domain composed of 10 amino acids is also completely conserved among these four *C. sporogenes* strains (Figure 1). Other partners of SpoIVA in the basement layer of the coat, which can be either SafA and SpoVID in *B. subtilis* or SipL in *C. difficile* (Touchette et al., 2019). These proteins contain a LysM domain (Touchette et al., 2019). BLAST searches revealed that there are no homologous proteins of *B. subtilis* SafA or SpoVID in *C. sporogenes*. There is one protein (GenBank: EDU37066.1) in *C. sporogenes* ATCC 15579 that contains a possible LysM domain. There are two homologous proteins of *C. difficile* SipL in *C. sporogenes* ATCC 15579: one (GenBank: EDU38021.1) shows 23% homology in total length with a possible LysM domain at the C-terminus, the other (GenBank: EDU37594.1) is homologous to only a portion of the LysM domain at the N-terminus. These results suggest either a poor level of conservation of these proteins among spore-formers or the existence of another family of proteins that functions to aid the transition from a MC-proximal cap to a full encasement of the spore coat. However, further research is required to completely elucidate the early stages of coat assembly in *C. sporogenes* and other *Clostridium* species, excluding those in the Peptostreptococcaceae family.

Compared to *C. botulinum*, *C. sporogenes* is a manageable bacterium owing to its lack of toxin production and low biosafety level. Moreover, *C. sporogenes* also shows a high spore formation rate, making it an excellent model for investigating sporulation. While the inactivation of the gene *csxA* in strain ATCC 15579 is important for exosporium assembly (Janganan et al., 2020), we focused on inactivating *spoIVA*, an essential gene for the formation of resistant spores in *C. sporogenes* NBRC 14293. This inactivation enabled comparative analysis with pathogenic bacteria such as *C. botulinum* and *C. difficile*. This study provides novel insights into spore morphogenesis in *C. sporogenes*. To identify specific spore-forming genes in *C. sporogenes*, it is necessary to obtain defective mutants through random mutagenesis and comprehensively characterize their traits, similar to the approach previously used in *B. subtilis*. Furthermore, comprehensive genetic analysis, such as the *B. subtilis* genome project, should be performed in the future.

## Data availability statement

The datasets presented in this study can be found in online repositories. The names of the repository/repositories and accession number(s) can be found in the article/Supplementary material.

## Author contributions

RK: Conceptualization, Formal analysis, Funding acquisition, Investigation, Writing – original draft, Writing – review & editing. BD: Writing – review & editing. IM-V: Writing – original draft, Writing – review & editing. HT: Conceptualization, Investigation, Writing – original draft, Writing – review & editing.

## Supplementary material

Supplementary Figure 1 Schematic of the construction of *C. sporogenes spoIVA* mutant. Oligonucleotides for PCR were designed to construct *spoIVA* mutants. To sequence *spoIVA*, a PCR fragment was subjected to TA cloning **(A)**. *spoIVA* of *C. sporogenes* NBRC 14293 was inactivated using the ClosTron mutagenesis system **(B)**. The pMTL007-CE2 ClosTron mutagenesis vector, an intron retargeting *spoIVA*, was used to generate pDIA6780 (pMTL007-CE2 Csp-*spoIVA-*160a). Plasmid pDIA6780 was then transferred to *C. sporogenes* NBRC 14293 via conjugation. *Clostridium sporogenes spoIVA* clones were selected on BHI agar containing erythromycin (2.5 μg/ml). To confirm the insertion of the group II intron into *spoIVA*, PCR was performed using two primer pairs: one flanking the integration site in *spoIVA* (RK1-RK8) and the second with a primer in *spoIVA* (RK8) and the intron (EBSu).

Supplementary Figure 2 Whole images from phase-contrast and fluorescence microscopy. *C. sporogenes* NBRC 14293 **(A,B)** and the *spoIVA* mutant **(C,D)** were cultured on GAM at 37°C for 24 h. Aliquots of the cells were stained with APBT. The cells were observed using phase-contrast microscopy **(A,C)** and fluorescence microscopy. Merged images are also shown **(B,D)**. Yellow arrowheads indicate vegetative cells. Light blue arrows indicate prespore and/or forespores, corresponding to stages II-III. Purple arrowheads indicate forespores in the mother cell, corresponding to stages IV–V. Green arrowheads indicate forespores in the mother cell, corresponding to stage VI. Orange arrowheads indicate free spores, corresponding to stage VII. Scale bars represent 5 μm.

Supplementary Figure 3 Transmission electron microscopy of the *spoIVA* mutant sporulating cells. The *spoIVA* mutant was grown on GAM at 37°C for 24 h after inoculation and analyzed using transmission electron microscopy. A sporulating cell is shown. Abnormal spore coats and/or exosporium structures (SC/EX) were observed in the sporulating cell. EX, SC, CT, C, SC, and MC represent the exosporium, spore coat, cortex, core, and mother cells, respectively. The sizes indicated with the scale bar are shown.

Supplementary Table 1 List of oligonucleotides used in this study.
