# Supplementary figures and images for: SpoIVA is an essential morphogenetic protein for the formation of heat- and lysozyme-resistant spores in Clostridium sporogenes NBRC 14293
Source: Front Microbiol. 2024 Apr 24;15:1338751. doi: 10.3389/fmicb.2024.1338751 (PMC11076785; doi:10.3389/fmicb.2024.1338751)

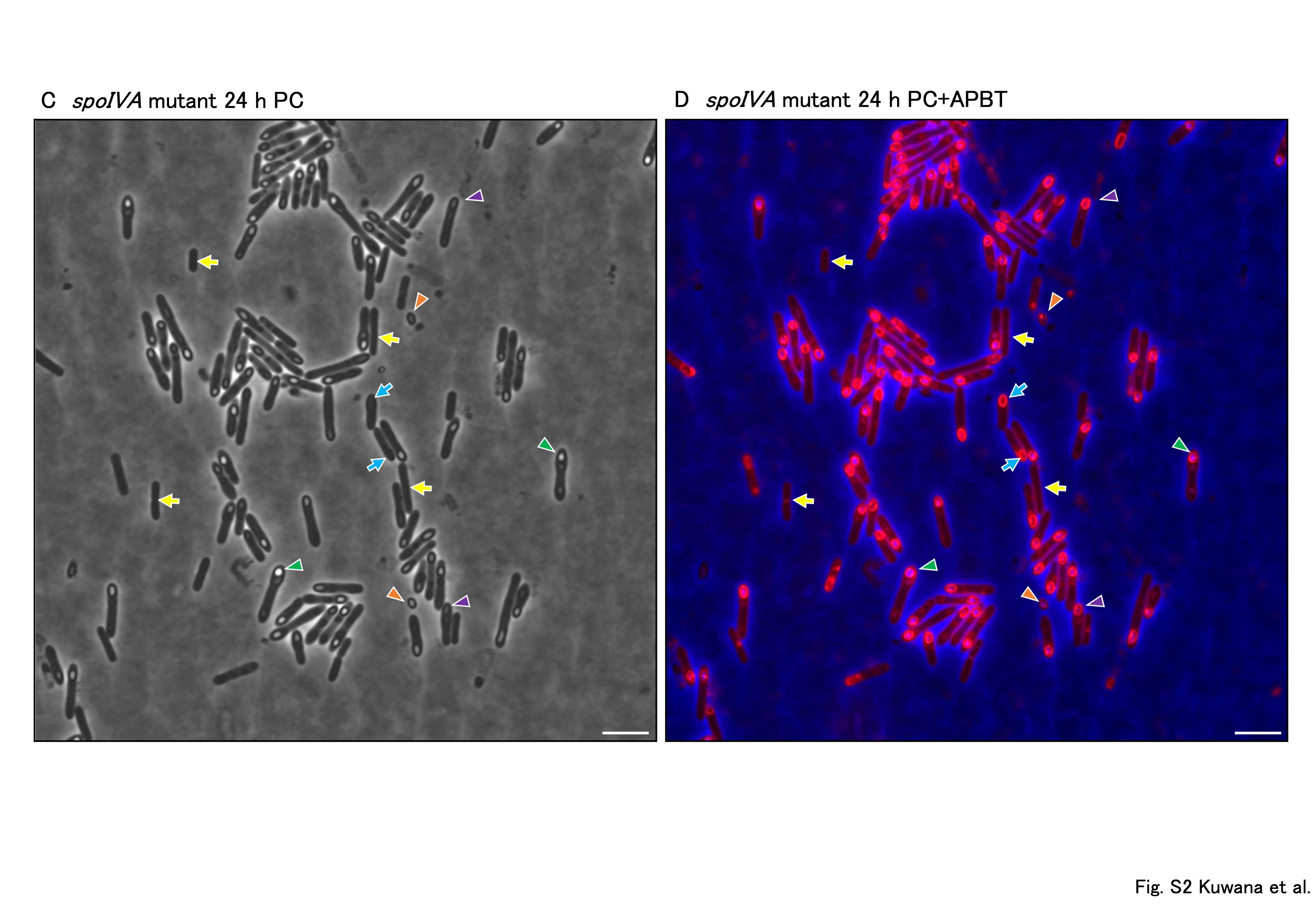

Supplement: Supplementary file 1 [file Image_3.jpeg]

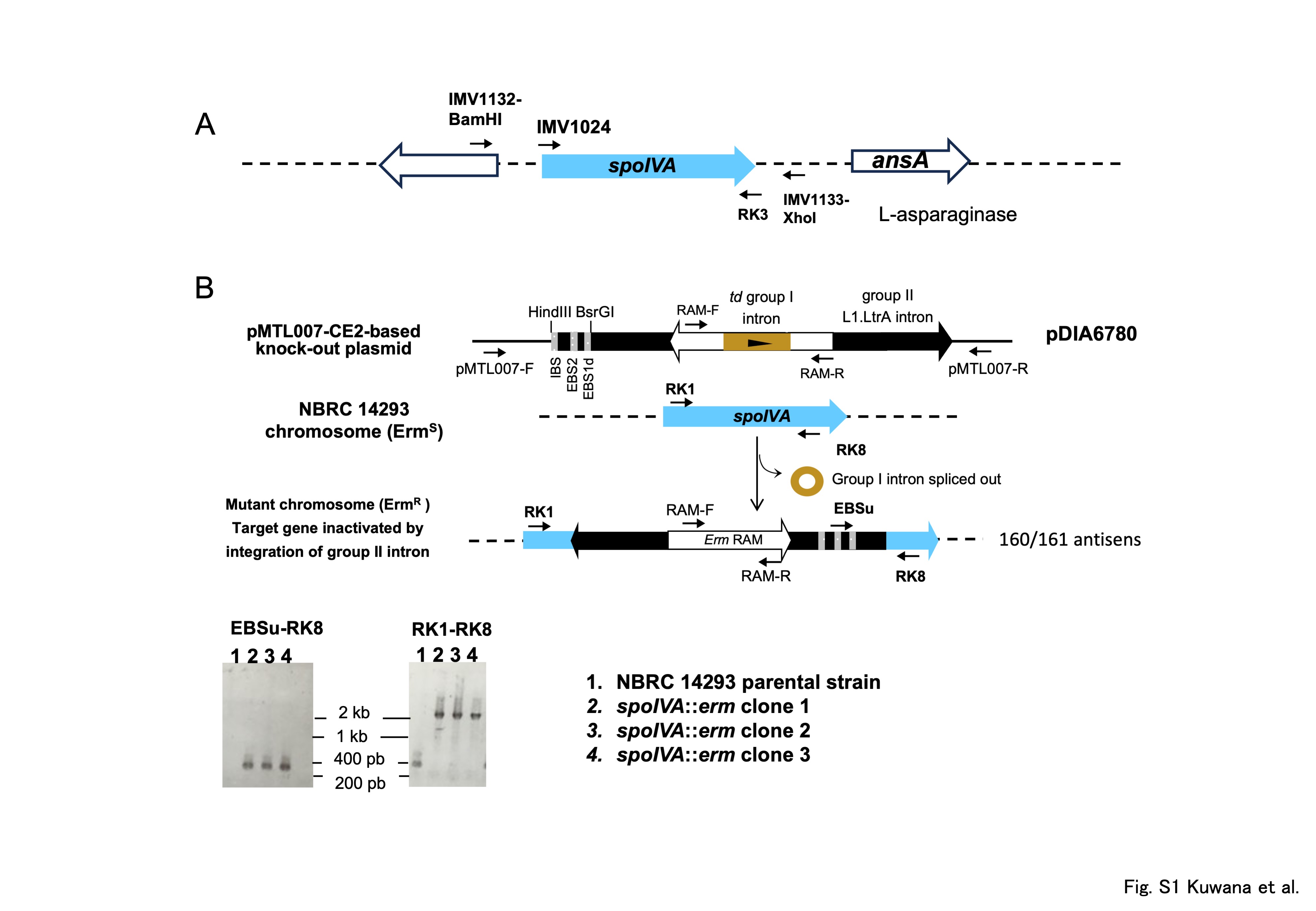

Supplement: Supplementary Figure 1 — Schematic of the construction of C. sporogenes spoIVA mutant. Oligonucleotides for PCR were designed to construct spoIVA mutants. To sequence spoIVA, a PCR fragment was subjected to TA cloning (A). spoIVA of C. sporogenes NBRC 14293 was inactivated using the ClosTron mutagenesis system (B). The pMTL007-CE2 ClosTron mutagenesis vector, an intron retargeting spoIVA, was used to generate pDIA6780 (pMTL007-CE2 Csp-spoIVA-160a). Plasmid pDIA6780 was then transferred to C. sporogenes NBRC 14293 via conjugation. Clostridium sporogenes spoIVA clones were selected on BHI agar containing erythromycin (2.5 μg/ml). To confirm the insertion of the group II intron into spoIVA, PCR was performed using two primer pairs: one flanking the integration site in spoIVA (RK1-RK8) and the second with a primer in spoIVA (RK8) and the intron (EBSu). [file Image_1.jpeg]

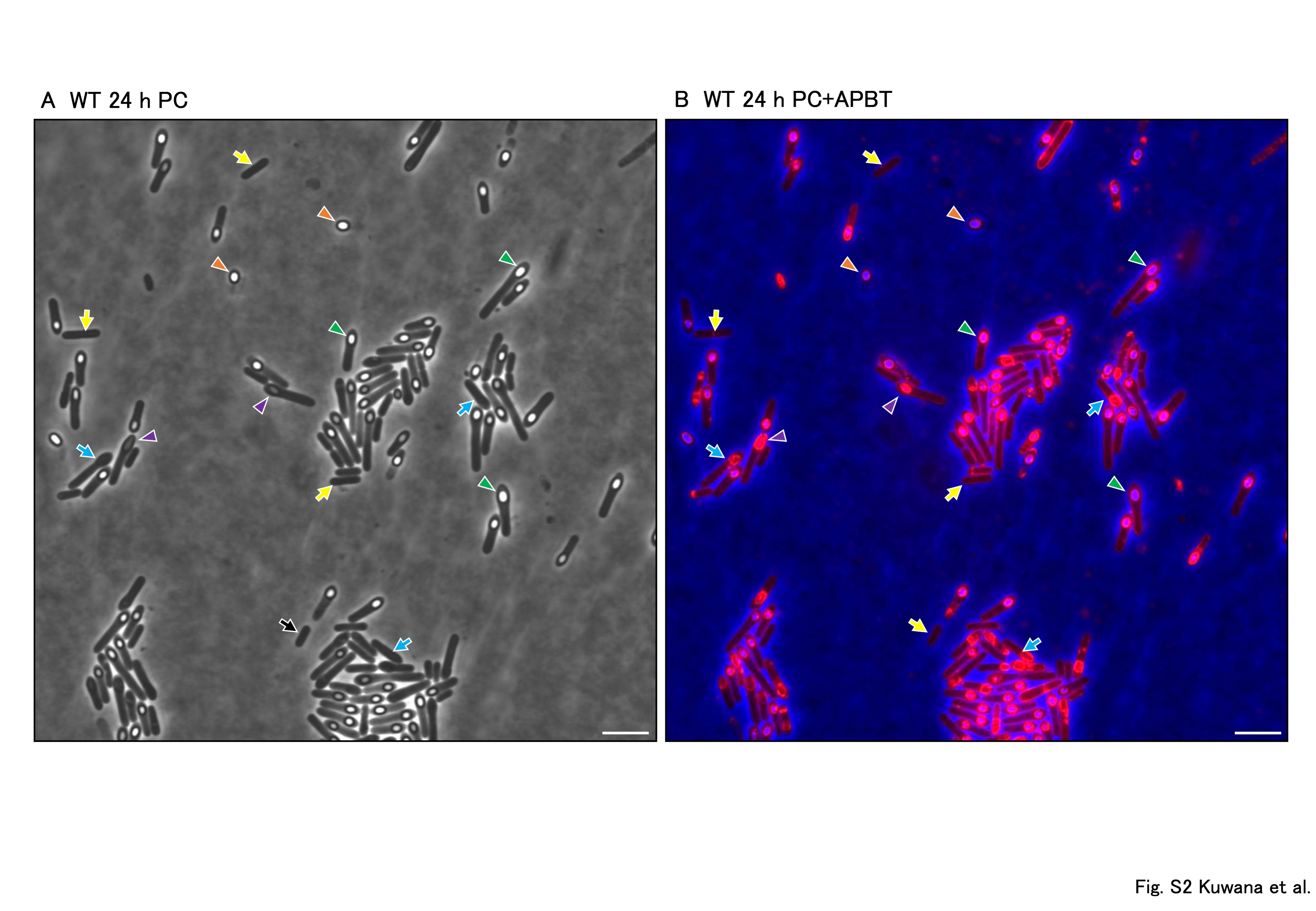

Supplement: Supplementary Figure 2 — Whole images from phase-contrast and fluorescence microscopy. C. sporogenes NBRC 14293 (A,B) and the spoIVA mutant (C,D) were cultured on GAM at 37°C for 24 h. Aliquots of the cells were stained with APBT. The cells were observed using phase-contrast microscopy (A,C) and fluorescence microscopy. Merged images are also shown (B,D). Yellow arrowheads indicate vegetative cells. Light blue arrows indicate prespore and/or forespores, corresponding to stages II-III. Purple arrowheads indicate forespores in the mother cell, corresponding to stages IV–V. Green arrowheads indicate forespores in the mother cell, corresponding to stage VI. Orange arrowheads indicate free spores, corresponding to stage VII. Scale bars represent 5 μm. [file Image_2.jpeg]

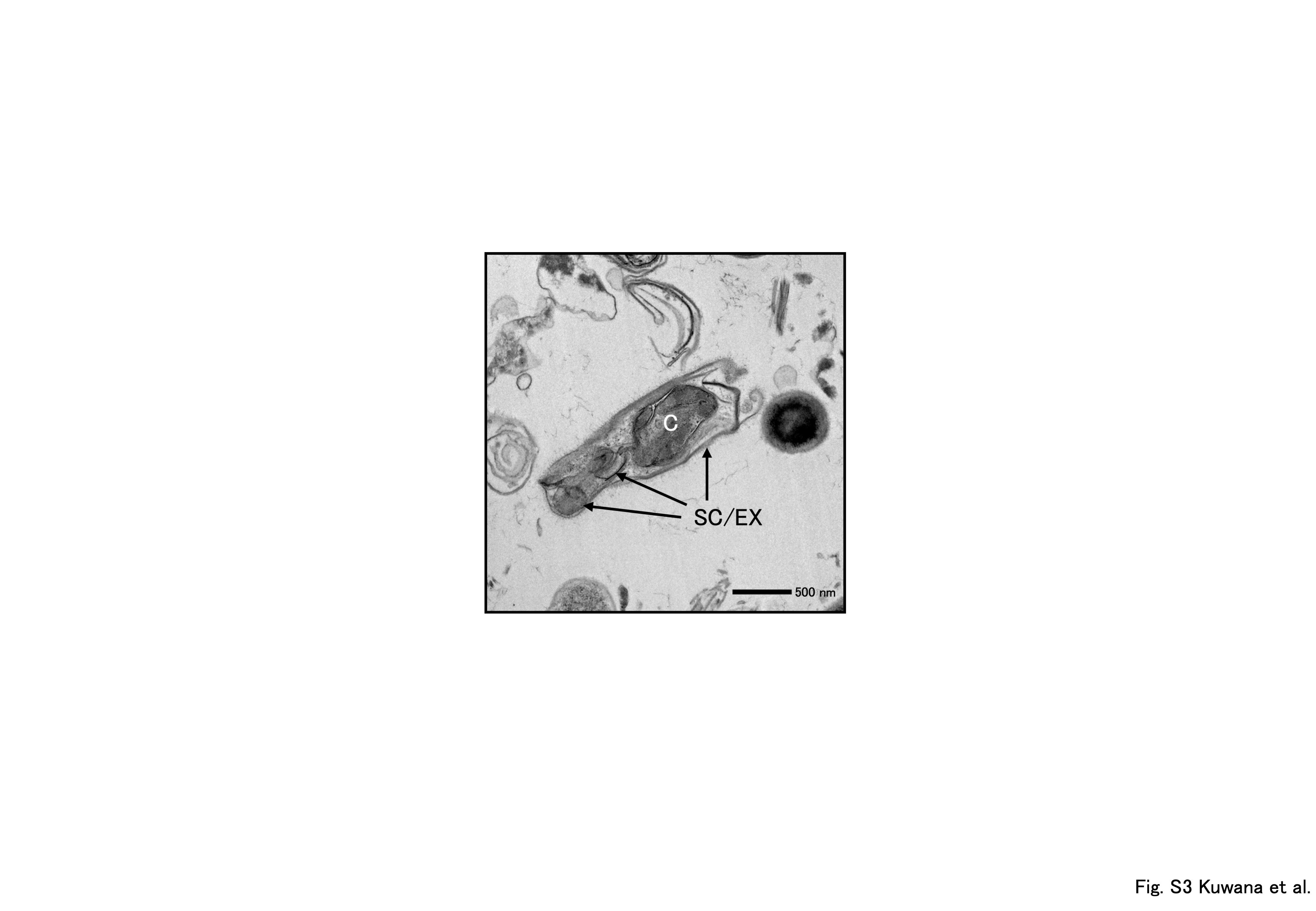

Supplement: Supplementary Figure 3 — Transmission electron microscopy of the spoIVA mutant sporulating cells. The spoIVA mutant was grown on GAM at 37°C for 24 h after inoculation and analyzed using transmission electron microscopy. A sporulating cell is shown. Abnormal spore coats and/or exosporium structures (SC/EX) were observed in the sporulating cell. EX, SC, CT, C, SC, and MC represent the exosporium, spore coat, cortex, core, and mother cells, respectively. The sizes indicated with the scale bar are shown. [file Image_4.jpeg]
