# Supplementary material for: SpoIVA is an essential morphogenetic protein for the formation of heat- and lysozyme-resistant spores in Clostridium sporogenes NBRC 14293
Source: Front Microbiol. 2024 Apr 24;15:1338751. doi: 10.3389/fmicb.2024.1338751 (PMC11076785; doi:10.3389/fmicb.2024.1338751)
Supplement: Supplementary Table 1 — List of oligonucleotides used in this study. [file Table_1.docx]

# Supplementary Table

**Table S1. List of oligonucleotides used in this study.**

| **Primer** | **Sequence** **(5' to 3')** | **Characteristics** |
| --- | --- | --- |
| IMV1024 | TTGGATAATTTTAATATATACAAAGATATCG | Conserved 5′ region of *spoIVA* |
| RK3 | TTATAATATGATACAGATAAGTCCTCCATTACC | Conserved 3′ region of *spoIVA* |
| IMV1132 | GGGGATCCTTGAGGGCCAGTAGG | 5′ *spoIVA* with a *Bam*HI restriction site |
| IMV1133 | CCGCTCGAGTTGCCTTTGTAACCACA | 3′ *spoIVA* with an *Xho*I restriction site |
| RK11 | AAAAAAGCTTATAATTATCCTTACATCTCTAGCCCGTGCGCCCAGATAGGGTG | SpoIVA-160\|161a-IBS primer |
| RK12 | CAGATTGTACAAATGTGGTGATAACAGATAAGTCTAGCCCTTTAACTTACCTTTCTTTGT | SpoIVA-160\|161a-EBS1d primer |
| RK13 | TGAACGCAAGTTTCTAATTTCGGTTAGATGTCGATAGAGGAAAGTGTCT | SpoIVA-160\|161a-EBS2 primer |
| EBSu | CGAAATTAGAAACTTGCGTTCAGTAAAC | Universal oligo for PCR ClosTron |
| RK1 | TTGGATAATTTTAATATATACAAAGATATCGCAGAAAGAAC | Upstream the intron insertion |
| RK8 | GGCTTATCCTCTTCCTCCATATAGCC | Downstream the intron insertion |
| pMTL007-R | AGGGTATCCCCAGTTAGTGTTAAGTCTTGG | Sequencing pMTL007 |
| T7 | TAATACGACTCACTATAGGG | PGEMTeasy |
| SP6 | ATTTAGGTGACACTATAGAATAC | PGEMTeasy |
